# Interoception in migraine is characterised by normal accuracy but altered sensibility and behaviour

**DOI:** 10.1038/s41598-025-31149-0

**Published:** 2025-12-05

**Authors:** Siobhan Jones, Jessica Komes, Ester Benzaquén, Quoc C. Vuong, William Sedley

**Affiliations:** 1 Translational and Clinical Research Institute, Faculty of Medical Sciences, Newcastle University Faculty of Medical Sciences, Newcastle upon Tyne, UK; 2 School of Psychology, Newcastle University Faculty of Medical Sciences, Newcastle upon Tyne, UK; 3 Biosciences, Newcastle University Faculty of Medical Sciences, Newcastle upon Tyne, UK

**Keywords:** Migraine, Interoception, Allostasis, Homeostasis, Interoceptive awareness, Heartbeat detection task, Neuroscience, Neurology

## Abstract

Migraine is typically precipitated by unaccustomed changes in one’s internal state and/or external environment. Migraine symptoms largely involve increased, noxious, awareness of bodily changes and external stimuli. Links have been proposed between migraine and interoception (sensing and interpreting internal states), but direct evidence is limited. Unmedicated, otherwise healthy, age-matched female participants were grouped by migraine tendency: control (no unprovoked headaches, n = 19); low-frequency ( < = 3 migraines/month, n = 20); high-frequency ( > = 4 migraines/month, n = 19). Interoception was assessed, interictally, with standardised questionnaires such as MAIA-2 and a widely used heartbeat counting task. The notable significant questionnaire-based difference was in the ‘noticing’, ‘not distracting’ domain of the MAIA-2; controls were least likely to continue activities despite experiencing physical discomfort, and high-frequency migraineurs most likely. Follow-up questioning clarified that this behaviour related predominantly to migraine-related symptoms. The heartbeat task found no differences in accuracy. Interoceptive accuracy scores were not reduced in migraine participants. Self-reported tendency to deliberately ignore physical discomfort, is associated with migraine frequency. Confidence (sensibility) showed a significant main effect of group. The low-frequency migraine group had the lowest confidence but interpretation of this finding requires further attention which could form a longitudinal study over the migraine cycle to distinguish causes from effects of migraine.

**Supplementary Information:**

The online version contains supplementary material available at 10.1038/s41598-025-31149-0.

## Introduction

Migraine is a common yet incompletely understood nervous system condition, affecting approximately 12% of the population^1^, with chronic migraine (CM) affecting 1–2%. It is likely that many more individuals with migraine are undiagnosed. In the UK around 10 million people aged 15–69 years old suffer with migraine^2^, with an annual healthcare cost of £150 million.

Migraine is typically characterised by a headache, often severe, and a noxious hypersensitivity to physical or mental activity and external stimulation (for instance light or sound); it can be highly disabling and can feature nausea and vomiting^3^. The migraine episode is divided up into four phases (not all of which are present in all individuals) comprising the premonitory phase characterised by autonomic and mood changes, (in some individuals) the aura phase characterised by focal neurological symptoms, the headache phase, and concluding with the post-drome phase^4^.

Because migraine triggers largely consist of unaccustomed alterations in physiological state, we have previously posed the question of whether migraine helps to stabilise internal bodily states when these are pushed beyond predictable limits^5^. Therefore, the question naturally arises of whether individual differences in people’s perception of internal states (interoception) are either of a cause and/or consequence of frequent migraines. Interoception is known to be linked to anxiety^6^, and migraine is known to be linked to anxiety^7^, so therefore it would be interesting to see if there would be differences in interoception between groups that were mediated by anxiety rather than being directly migraine-associated.

### Interoception

Interoception strictly means perception or sensing of the state of the body^8^, for instance heartbeat, respiration and hunger, and is vital for survival in informing the nervous system about the body’s physiological needs. It also encompasses the ability to interpret and respond appropriately to patterns of internal signals. Interoception constitutes the afferent limb of allostasis (maintenance of physiological states across changing situations and requirements) and interrelates with cognition/psychology and in particular, emotional regulation)^9^. Interoceptive experience is influenced by both descending predictions about the expected state of the body and ascending visceral sensations^10^.

### Measuring interoception

Measurement of interoception can be divided into 3 domains: *interoceptive accuracy* (objective performance), *interoceptive sensibility (confidence)* (self-reported beliefs about the strength with which interoceptive signals are perceived) and *interoceptive awareness* (the correspondence between *accuracy* and *sensibility*; i.e. being more accurate when one thinks they are being more accurate, and vice versa)^11^.

Aspects of interoceptive sensibility are measured by questionnaires, and subjective self-report measures such as confidence performing an interoceptive task^12^.

There are also more detailed classifications for individual differences in interoception. The 3 domains mentioned above, also referred to as the classic tripartite classification^12^, is what we focussed on, for its simplicity and familiarity. However, we recognize there is more complexity to interoception, including different facets of sensibility^13^, and likewise aspects of interoception that are beyond this tripartite classification.

Recent studies concluded that there is not one optimal questionnaire that covers all aspects of interoception, and we aimed to explore a range of facets of interoceptive sensibility for their potential role in migraine tendency.

The ability to monitor one’s own heartbeats has emerged as a potential biomarker in some neurological and psychiatric disorders^14^. Garfinkel et al., (2015) employed the heartbeat tracking task to determine individual differences in interoceptive accuracy. Research using heartbeat detection (HBD) has helped to shed light on key processes that relate to interoception.

### Links between interoception and migraine

#### Clinical overlap between migraine and interoception

Migraine is often considered within the wider context of physiological regulatory systems, i.e. homeostasis and allostasis, which rely on interoception as their main sensory input^5^. Furthermore, between individuals, there is a strong correspondence between which sensory modalities act as triggers (e.g. hunger) and manifest premonitory symptoms (e.g. food cravings)^15^, to the point that it can be difficult to assign a particular symptom to one category or the other^16^.

#### Migraine and allostasis

Early concepts of homeostasis (maintaining rigid physiological stability) are inherently inefficient and have since been expanded and replaced by ‘allostasis’, defined as achieving “stability through change”^17^. More specifically, allostasis describes the process whereby organisms change the defended levels of regulated parameters as needed to adjust to new or changing environments, including pre-emptive responses to anticipated changes^17^.

Allostasis relies heavily on predictions, whose accuracy is indicated by the magnitude of ‘prediction errors’, which signal discrepancies between predicted and sensed physical states. For allostasis to function efficiently and safely, its underpinning predictions must be sufficiently accurate. We have recently characterised migraine as, in its ‘intended’ form, as an adaptive process to restore allostatic accuracy in the face of persistently elevated interoceptive prediction errors (IPE)^5^. In this account, elevated IPE is the cause of migraine, amplification of IPE is the mechanism of migraine (leading first to the premonitory phase, then the migraine attack phase), and withdrawal from activity and stimulation is the forced behaviour that promotes stability to restore the accuracy of predictions. However, failure to respond to migraine episodes by reducing IPE, or external or internal environments that present ongoing unavoidable sources of error, is thought to lead to persistent, recurrent, and self-reinforcing amplification of IPE, clinically causing high-frequency episodic or chronic migraine, and physiologically leading to excessive stress responses that result in a kind of permanent damage termed ‘allostatic load’^18^.

#### Migraine as a condition of interoception

The idea that migraine is primarily a condition of altered interoceptive processing sits well within its existing categorisation as “a disorder of sensory processing”^3^. Migraine may also be a phenomenon that straddles multiple categories, being adaptive in some instances and disordered in others (much like pain)^8,24^. Interoceptive abilities could therefore be a key factor in determining the susceptibility to, and the frequency/chronicity of, migraine. For instance, greater interoceptive accuracy might help to prevent migraines by reducing IPE themselves, and/or through the formation of more accurate predictions of future physiological states^5^. Additionally, or alternatively, greater interoceptive sensibility (the subjective strength of interoceptive signals and appropriate appraisal) could result in fewer migraines (if IPE are highlighted and addressed earlier) or more migraines (if amplified IPE cross the threshold to trigger migraines more often). Furthermore, behavioural responses to interoceptive signals may be important, for instance whether an individual acts on or ignores a particular sensation, and this in turn may depend upon metacognitive interoceptive awareness. Finally, in the opposite direction of causation, migraine episodes might cause lasting alterations in interoception, for instance as a partial carryover of the noxiously increased interoceptive signals experienced during migraine attacks.

#### Aims and hypotheses

Migraine and interoception is a largely unexplored area but its importance is starting to be recognised^19^. We conducted a study to ascertain abnormalities of interoception as a predictor of migraine frequency by employing a Heartbeat Tracking Task and objective and subjective measures of interoception in the form of questionnaires in order to capture the three interoceptive domains.

In line with our overarching hypothesis that migraine is both a response to excessive interoceptive prediction errors and a mechanism that amplifies interoceptive signals, we predicted several relationships between migraine frequency and interoception that we might see to reflect aspects of this bidirectional relationship:

1. Low interoceptive accuracy might be associated with greater tendency to migraine, because low accuracy would result in higher rates of prediction error (mismatch between predicted and sensed bodily states).
2. High interoceptive sensibility (confidence) might be associated with greater tendency to migraine, for one of two reasons: firstly because high sensibility indicates interoceptive signals being felt more strongly, meaning error signals could more easily cross the threshold to trigger migraine; secondly because migraine could have a lasting effect on amplifying interoceptive signals, leading to increased sensibility in proportion to the frequency of migraines.
3. Low interoceptive sensibility might be associated with a higher frequency of migraines, if it associated with a tendency for interoceptive errors to go relatively unnoticed, hence unaddressed, until a late stage around when migraine is triggered.

## Methods

### Participants

We recruited age-matched groups of female participants aged between 18 and 65, stratified by self-reported migraine frequency, into Low Frequency (LF), High Frequency (HF) and Control groups (Ctrl). Due to such a challenging criterion of unmedicated matched groups differing only in migraine frequency, recruited to a target of 20 participants per group, based on pre-screening and pre-enrolment matching of age within groups. Adverts for the study were posted on the university website, a migraine charity website and Google Ads. Interested participants would get in touch via email after which they were asked to complete a pre-screen questionnaire which determined whether they fulfilled criteria. They were also sent additional information regarding the study. Once the completed pre-screen questionnaires were returned those successful would be contacted with the offer of being involved in the study and the those who did not fulfil criteria were also informed. Out of 170 pre-screened individuals, 112 did not fulfil criteria as they were either on regular preventative treatment and/or suffered with comorbidities. On attendance in person, the lead researcher took a detailed case history to confirm the diagnosis of migraine according to the ICHD-3 criteria^20^ and estimate the number of monthly migraine days, headache days, and presence or absence of aura.

The experiment was conducted on interictal days (defined as more than 24 h since most recent headache, to avoid postdrome changes, and more than 24 h prior to subsequent migraine, to avoid inadvertently capturing premonitory changes). Because of this requirement, an exclusion criterion was headache or migraine symptoms ‘on most days’, to the point where satisfying the interictal criteria would be difficult. As these might in principle affect interoception, participants were excluded who were taking any migraine preventative medication, or any regular medication with a known action on the central, peripheral, or autonomic nervous system, including sedatives, antidepressants, antihypertensives and anticonvulsants. Other exclusion criteria included any known abnormality of brain structure (e.g., stroke, tumour), or other neurological disorder (e.g., multiple sclerosis or epilepsy), psychiatric comorbidity of sufficient severity to limit certain activities of everyday life, or abnormality of cardiac rhythm or function. Participants were also advised not to attend if they had taken a triptan in the last 24 h. The Ctrl group criteria included not experiencing headaches ever except during physical illness. The rest of the exclusion criteria were the same as the migraine participants. In the migraine groups the participants were also asked to notify the researcher if they suffered a migraine 24 h after the testing. The protocol was to exclude data from these participants, but no participants reported a migraine in the 24 h after testing.

We did not achieve 20 in each group. The LF group comprised 20 participants with LF migraine (≤ = 3 migraine days per month), the HF group comprised 19 participants with HF migraine (≥ = 4 migraine days per month), and the Ctrl group comprised 19 participants with no history of unprovoked headaches (even reports of ‘tension headache’ was an exclusion criterion in case it reflected a limited or milder form of migraine). The threshold of 4 migraine days per month was chosen to give the most bimodal distribution of monthly migraine days across screened participants. It also accords with the typical threshold at which prophylactic treatment would be offered. Ethical approval was provided by Newcastle University Ethics Committee (Ref: 37672/2024). Informed written consent was obtained from all participants. All methods were performed in accordance with institutional procedures and guidelines, and with General Data Protection Regulation (GDPR), Good Clinical Practice (GCP) for research and the Declaration of Helsinki.

### Case history and questionnaires

Once screened for eligibility and enrolled, participants were sent the following validated mood and behavioural questionnaires via an online link to complete prior to attendance for laboratory testing:

- Body Perception Questionnaire (BPQ) short form^21^.
- Migraine Specific Quality of Life Questionnaire (MSQL)^22^.
- Hospital Anxiety and Depression Scale (HADS)^23^.
- Intolerance of Uncertainty Scale (IUS)^24^.
- Multidimensional Assessment of Interoceptive Awareness (MAIA-2)^25^.
- Inventory of Differential Interoceptive Awareness-Short form (IDIA)^26^.

There are many questionnaires which assess self-reported interoceptive traits and behaviours, which provide a more granular view of sensibility, as well as encompassing aspects of interoception beyond the tripartite classification. Recent studies concluded that there is not one optimal questionnaire that covers all aspects of interoception. The BPQ questionnaire is frequently used questionnaire for measuring interoception described as a ‘subjective experience of information arising from within the body’^27^ for example perceiving interoceptive signals in everyday situations such as “how fast am I breathing”^21^. The MAIA-2 captures the perceived ability of sensing signals originating from within the body (such as noticing, attention regulation, emotional awareness, self-regulation, body listening, and trusting^27^. The IDIA questionnaire is inspired from the MAIA-2 by using body information and breaking this down into positive or negative signals^26^. The HADS questionnaire is a commonly used screening tool in the headache clinic to monitor depression and anxiety. If either comorbidity is recognised early, it can increase the chances of better responses and engagement to migraine treatment plans^28^. The IUS questionnaire looks at transdiagnostic factors across psychopathology such as anxiety, worry and fear^29^.

### Follow−up questionnaires

We performed an exploratory principal component analysis (PCA) to understand the factor structure of the various questionnaires and their domains (see supplementary information 1). This revealed a significant uncorrected main effect of group in a factor representing the ‘not -distracting’ and ‘noticing’ domains of the MAIA2. In itself, this result would not differentiate migraine-related from non-migraine related discomfort, nor the noticing of that discomfort from the behavioural response to it. We were therefore inspired to perform follow-up testing that explicitly differentiated migraine from non-migraine discomfort and noticing from not distracting. Due to concerns about the stability of the PCA with relatively small sample sizes, and that our results led to the same conclusions without using a PCA, we report in the main text only the non-PCA-based analyses.

To explore relevant differences specifically relating to migraine or non-migraine symptoms, we sent follow-up questionnaires to all participants that re-asked questions relating to (uncorrected) significant main effects of group, specifically asking ‘relating to migraine symptoms’ or ‘excluding migraine symptoms’ for each question in the MAIA2 ‘not - distracting’ and ‘noticing’ domains.

We re-tested identical questions of the ‘not-distracting’ domain e.g. (“I distract myself from sensations of discomfort”), and the ‘noticing’ domain e.g. (“when I am tense I notice where the tension is located in my body”), with each of two clear prefaces: ‘relating to migraine symptoms’, or ‘excluding migraine symptoms’. The Ctrl group were re-questioned without this differentiation, to maintain parity. The follow-up questionnaires were only answered by a subset of participants (9 HF, 14 LF and 15 Ctrl).

### Heartbeat tracking task

Participants performed the heartbeat tracking task, implemented in Matlab^30^ using the Psychtoolbox toolbox^31^, in a dimly lit sound-attenuated room^12^. Participants were asked to count heartbeats by only concentrating on their body and not by taking their pulse or using any exteroceptive cues. The task simply required them to count the number of heart beats occurring during a specified time interval. There were 20 trials (with 1 extra practice trial), which differed in length, in randomised order, and were indicated with auditory stimuli (“Start” and “Stop”) presented with free-field speakers. Four repetitions of each of the following durations were included: 17 s, 22 s, 27 s, 37 s, 47s. At the end of each trial participants were asked the on-screen question ‘How many beats did you count?’, followed by a second question of ‘How confident are you?’ (in their response being accurate) and given a scoring scale between 0 and 100 with ‘0’ equating to not confident, and with 100 being very confident^12^. The reported number of beats was then compared to the actual number of beats as extracted from the ECG monitor using a Biosemi ActiveTwo system^32^. ECG was recorded from the left wrist, and heartbeats counted using the Pan-Tompkin algorithm^33^, with manual confirmation of accuracy of fit for each trial. Accuracy and awareness scores were measured by examining the relation between the participants perception (self-reported account of their heartbeats) in comparison with the actual recorded heartbeats. Analysis was performed off-line, and participants were not given feedback about their performance. The accuracy was calculated as 1 - |a-p|/((a + p)/2) where ‘a’ is the actual number of heartbeats, and ‘p’ is the perceived number of heartbeats, i.e. one minus the absolute difference between actual and perceived number of beats divided by the mean of the actual and perceived number of beats. Awareness was calculated by the strength of linear correlation, across trials, between accuracy and confidence, expressed as Pearson’s r. Informal feedback received from the participants revealed that they did not rely on own beliefs of resting heart rate which is what several studies have suggested is how participants may undertake this task^34^ One-way ANOVA saw no significant difference in average resting heart rate over all groups.

### Statistical analysis

All analyses were performed using the SPSS software^35^. One−way ANOVA was used to detect main effects of participant group for each questionnaire, or each domain of multi-domain questionnaires. This was followed by pairwise T tests as post-hoc tests in cases where there was a main effect of group. As the heartbeat task comprised a small number of measures that were each the subject of prior hypotheses, these data were not corrected for multiple comparisons. As the questionnaire aspect of the study was exploratory, all questionnaire-based analyses were Bonferroni corrected for the total number of comparisons performed (primary and post-hoc) across both rounds of questionnaires (the original and follow-up).

**Table 1:** Group characteristics Fifty-eight age-matched female participants were grouped by migraine tendency: control (no unprovoked headaches, *n* = 19); low-frequency (< = 3 migraines/month, *n* = 20); high-frequency (> = 4 migraines/month, *n* = 19). Convention for numbers = mean (SD).

Group	*n*=	Age	Monthly migraine days	Monthly headache days	*n* with aura
High frequency	19	40 (14.4)	9 (5)	11 (6)	12
Low frequency	20	40 (13.9)	2 (1)	5 (4)	13
Control	19	40 (15)	N/A	N/A	N/A

## Results

Subject characteristics are summarised in Table 1.

### Heartbeat tracking task results

**Fig. 1:**
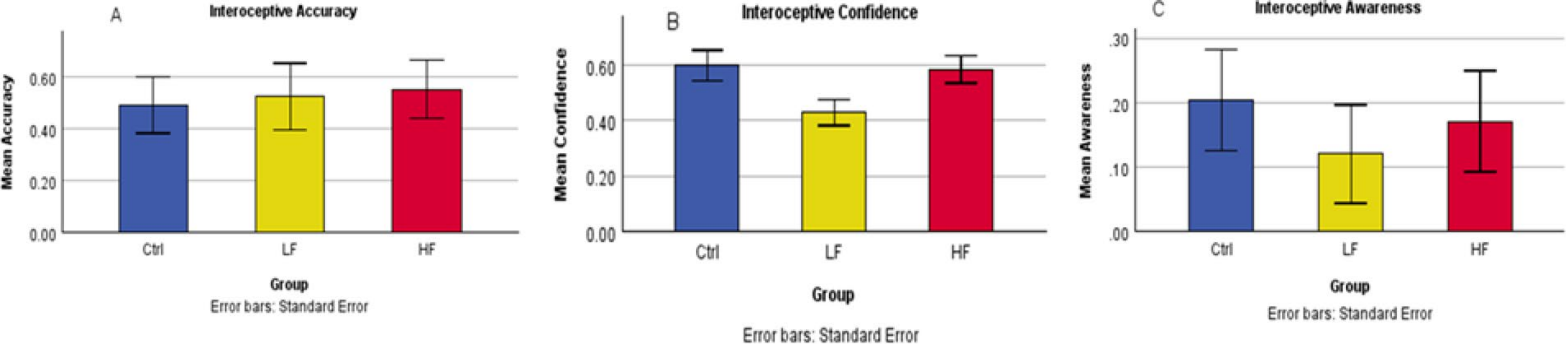
Results of the heartbeat counting task.Interoceptive accuracy (**A**) showed no significant effect of group. Interoceptive confidence (**B**) showed a significant main effect of group, but post-hoc tests did not show significant differences between groups after Bonferroni correction. Interoceptive awareness (**C**) was not significantly different between groups.

The one-way ANOVA analyses for main effect of group (Fig. 1A) showed no difference in interoceptive accuracy (F = 0.293, *p* =.747), whilst interoceptive confidence (Fig. 1B) showed a significant difference (F = 3.512, *p* =.037). Interoceptive awareness (Fig. 1C) was not significant (F =0.294, *p* =.746). For interoceptive confidence Cohen’s d was as follows: Ctrl vs. HF = 0.67, Ctrl vs. LF = 0.751, HF vs. LF = 0.727. The Bonferroni-corrected post-hoc comparisons did not reach statistical significance (Ctrl vs. HF *p* = 1.000, HF vs. LF *p* =.103, LF vs. Ctrl *p* =.062)

### Questionnaire responses

**Fig. 2:**
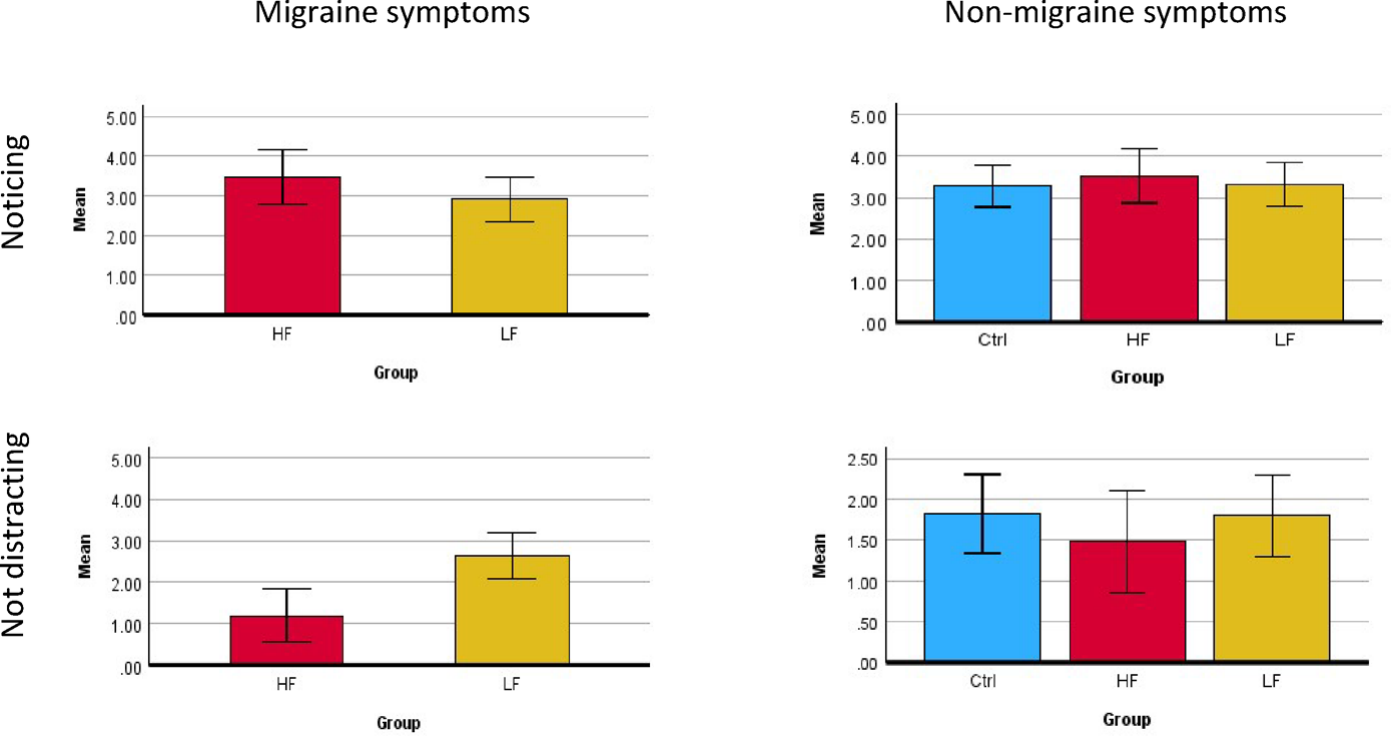
Follow−up questionnaires of the MAIA sub-scales ‘noticing’ and ‘not distracting, relating to migraine-related and non-migraine-related symptoms only. There was a significant difference between HF and LF groups only for the ‘not distracting’ sub-scale with migraine symptoms (p = 0.038) (Bonferroni-corrected for 19 group comparisons). Error bars represent standard error of the mean.

Results for the initial questionnaire data are show in Table 2. Only the MAIA2 ‘not distracting’ domain showed an uncorrected main effect of group (*p* =0.05). As described in Methods, we were motivated to distinguish migraine-related from non-migraine-related symptoms where potentially relevant to significant or near-significant group differences. There were no significant differences in non-migraine related symptoms as a main effect of group (F = 0.711, *p* =4.60). The ‘not distracting’ domain relating to migraine symptoms showed significantly lower scores (indicating a greater tendency to persevere despite migraine-related discomfort) in the HF than the LF migraine group (F = 12.342, *p* =0.02), whilst the ‘noticing’ domain showed no differences between HF and LF groups (F = 148, *p* =2.00) (Fig. 2).

**Table 2:** Scores for each questionnaire domain for each group, from the main set of questionnaires (excluding follow-up questioning), and associated uncorrected p values for main effects of group from a one-way ANOVA with bonferroni correction, no main effects of group remain significant.

Questionnaire	*p* value	Mean (Ctrl)	Std error (Ctrl)	Mean (HF)	Std error (HF)	Mean (LF)	Std error (LF)
BPQ: Supradiaphragmatic reactivity	0.703	21.842	1.606	22.000	1.650	23.550	1.566
BPQ: Body awareness	0.385	64.789	4.138	59.111	4.251	56.950	4.033
BPQ: Subdiaphragmatic reactivity	0.876	10.158	0.792	10.667	0.813	10.650	0.772
IUS	0.297	25.421	1.924	26.944	1.977	29.600	1.875
MAIA2: Noticing	0.349	2.961	0.225	3.389	0.231	3.000	0.219
MAIA2: Not distracting	0.005	2.711	0.237	1.556	0.244	2.175	0.231
MAIA2: Not worrying	0.340	2.958	0.208	2.689	0.214	2.530	0.203
MAIA2: Attention regulation	0.388	2.753	0.200	2.913	0.206	2.523	0.195
MAIA2: Emotional Awareness	0.435	3.042	0.215	3.322	0.221	3.420	0.209
MAIA2: Self-regulation	0.669	2.855	0.257	2.583	0.264	2.563	0.251
MAIA2: Body listening	0.743	2.596	0.275	2.463	0.283	2.300	0.269
MAIA2: trusting	0.773	3.404	0.260	3.166	0.267	3.184	0.253
IDIA	0.106	2.131	0.166	2.408	0.170	1.899	0.162
HADS-A	0.296	7.316	0.652	8.778	0.670	8.200	0.636
HADS-D	0.797	13.105	0.641	12.611	0.658	12.550	0.624

## Discussion

### Interoceptive accuracy in migraine

Contrary to our original hypothesis, interoceptive accuracy was not reduced in participants with migraine. This null result is informative, as it contrasts with findings from other chronic pain populations, where lower interoceptive accuracy has been reported and is often negatively correlated with symptom severity^36^. The absence of such an effect in migraine highlights that interoceptive processes may not generalise across pain conditions and suggests a more nuanced relationship between interoception and migraine. Importantly, this observation is limited to the interictal phase; fluctuations in interoceptive accuracy across the premonitory or ictal stages remain to be explored.

These results align with recent work examining interoception and dissociation in migraine subtypes, which similarly suggest that the interplay between migraine and interoception is complex and may not conform to deficit-based models^37^. The inclusion of null results such as these is valuable for theory-building: rather than supporting a simple deficit hypothesis, they point towards a differentiated framework where interoceptive changes may be phase-specific or subgroup-dependent. Findings can also be considered in relation to recent neuroimaging study evidence^38^ looking at altered amygdala connectivity associated with reduced interoceptive accuracy in migraine. At first glance, this contrasts with our behavioural results showing no reduction in accuracy. However, these differences may reflect methodological and contextual factors. The study focused on chronic migraine and highlighted altered brain network connectivity, which may represent a neural vulnerability not always expressed behaviourally in interictal performance. Our null results may therefore reflect phase-dependent preservation of accuracy, or heterogeneity within the migraine population, whereas connectivity alterations could indicate latent mechanisms that manifest only under specific conditions (e.g., during attacks, or in more severely affected subgroups).

### Interoceptive confidence

In terms of interoceptive confidence, we observed a significant main effect of group, with the lowest mean scores in the low-frequency migraine subgroup. However, post-hoc tests did not reveal clear pairwise differences, which substantially limits the robustness of this finding. Moreover, the overall pattern was not consistent with any single hypothesis, indicating that the relationship between confidence and migraine frequency may be more complex than initially assumed. While this main effect is noteworthy, the null post-hoc outcomes are equally important in delineating the boundaries of interpretation.

Taken together, the findings suggest that interoceptive processes in migraine cannot be reduced to a straightforward deficit model. The preservation of interoceptive accuracy, alongside tentative but inconclusive group differences in confidence, underscores the need for future research employing longitudinal and phase-specific designs. Tracking both accuracy and confidence across the migraine cycle — and in relation to dissociation, affective symptoms, and attack predictability — could help clarify whether interoceptive changes represent causes, consequences, or correlates of migraine. By giving due attention to both significant and null findings, our results contribute to refining the theoretical models of interoception in migraine and highlight directions for future investigation.

### Tendency to notice and ignore physical discomfort is associated with higher migraine frequency

Questionnaire responses indicated that the HF migraine group reported a tendency to modify or reduce their activity less as a result of currently experienced migraine-related discomfort (scoring lower on the ‘not distracting’ subscale of MAIA2, specifying migraine-related symptoms specifically).

Conversely, there was no corresponding difference in the self-reported noticing of migraine-related discomfort (‘noticing’ subscale of MAIA2). Furthermore, there were no significant group differences in any of the questionnaire measures of interoception, anxiety, depression, or intolerance of uncertainty.

**Fig. 3:**
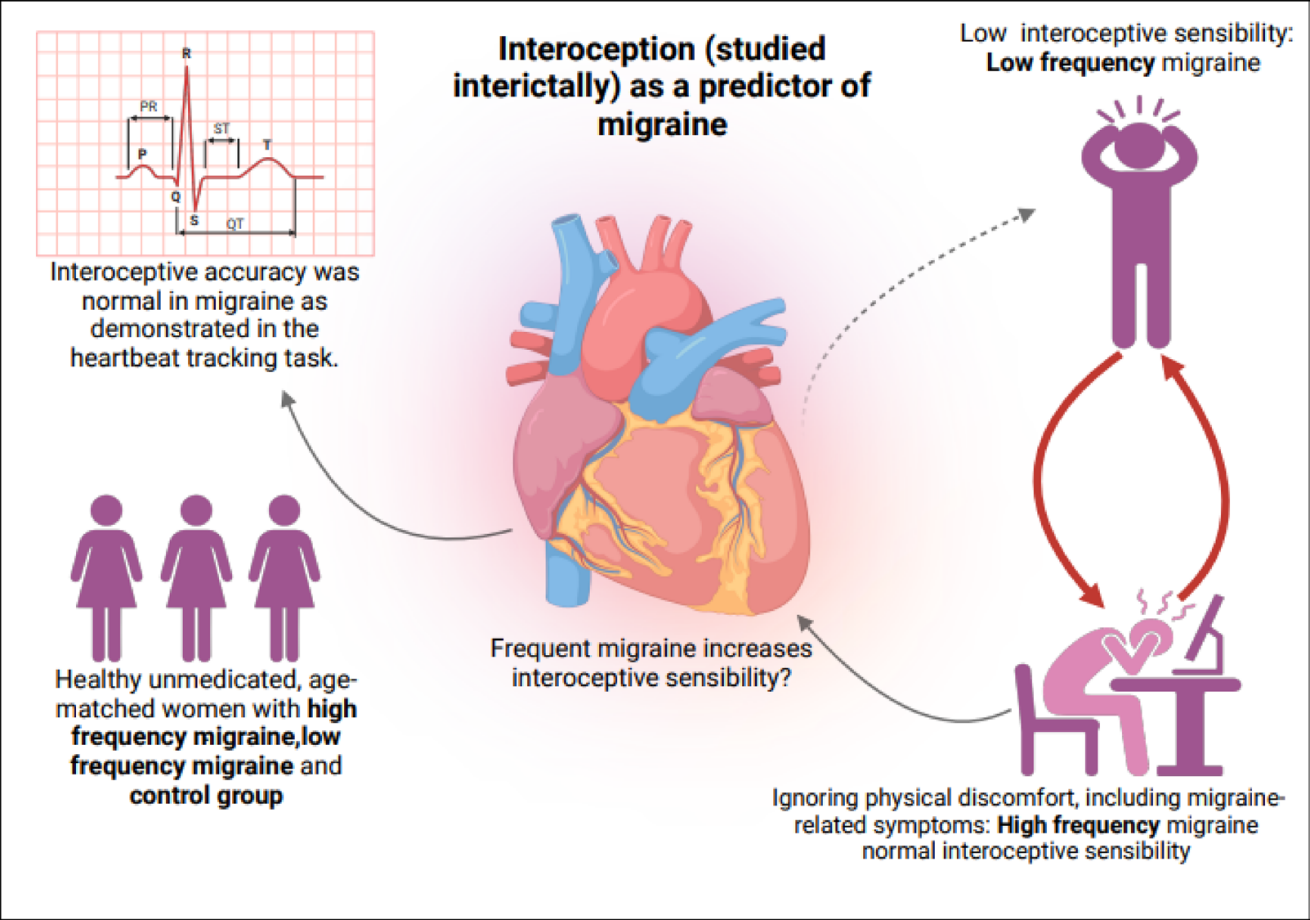
Schematic summary of interoception (studied interictally) as a predictor of migraine frequency.

A proportion of the difference in ‘not distracting’ scores might still have reflected living with an increased burden of migraine symptoms, despite no differences in the ‘noticing’ domain. However, it seems more likely that there is a specific association with self-reported tendency to ignore or ‘push-through’ migraine-related symptoms. This would be similar to our hypothesis 3, in that failure to change behaviour would hinder the utility of migraine to resolve interoceptive errors, with the difference that the problem is not with the awareness of the errors (as we had hypothesised) but rather that errors are more likely to go unaddressed even after causing migraine symptoms. If we accept the notion that the ‘mechanism’ of migraine is to increase the strength of interoceptive signals (i.e. interoceptive sensibility) in order to enforce behaviour that resolves errors, but these signals are then ignored by the individual continuing activities despite feeling bodily discomfort, then this might lead to even more migraines as a protective reflex (and a positive feedback loop ensuing) as illustrated in Fig. 3. This notion aligns well with the existing concept of ‘error awareness’ in migraine, in which repeated migraine episodes lead to a lasting increase in the awareness of interoceptive errors, which become aversive on account of being associated with migraine symptoms^39^.

We also consider the ‘not- distracting’ results through the lens of *fear avoidance*, which is often associated with chronic pain^40^. The HF participants in our study report to push on with activities despite pain. This could be construed as the opposite of fear avoidance, by persevering with activities known to cause discomfort. Alternatively, continuing activity could be also viewed as an avoidance tactic in distracting from physical discomfort.

### Strengths, limitations and future directions

This has been a specific study on ‘ordinary’ migraine, unconfounded by sex/gender, medication, or physical or mental health comorbidity. It has allowed us to be able to isolate only interictal differences, to avoid confounding immediate effects of migraine processes. We excluded chronic migraine and the more complex patients typical of tertiary headache clinics, where migraine is more ‘disordered’.

Limitations of the study included the relatively low number of participants, examining only one interoceptive domain objectively, and not longitudinally examining the immediate and short-term effects of migraine on interoception. One must take into consideration the measures of interoceptive ability and how they may differ due to required cognitive effort, and on the nature, length and the difficulty of the task^41^. Whilst the method of the heartbeat tracking task has been under scrutiny it has still been seen as a valid way of assessing interoceptive accuracy^34^. It is important for future studies to use other interoceptive tasks and/or modalities to ensure the findings generalise. Assessing separate interoceptive domains such as respiration or gastric interoceptive awareness^42^ could help address technical concerns, and search for generalisation of findings^43^.

Future directions could look at chronic migraine and also how migraine interrelates to commonly co-morbid conditions, such as chronic pain, in which interoceptive accuracy deficits have been observed^40^.

Our results also highlight the potential insights possible through questionnaire/subjective measures of interoception, not all relying on lab-based tests of accuracy, which opens the door to large-scale online questionnaire studies and incorporation into clinical consultations. Longer-term aims are to use this information to develop innovative interventions such as interoceptive training programmes to help prevent the conditions that cause migraine episodes from occurring.

## Conclusions

These data offer insight into the role that migraine tries to play in helping us safely regulate our physiology, lending some empirical support to our recently proposed model of migraine as a ‘failsafe’ to stabilise homeostasis/allostasis, and may help lead to a conceptual framework for patients to help them understand and non-pharmacologically manage their migraine by addressing its underlying ‘causes’. This study has provided some new interesting and theoretical findings in the relationship between interoception and migraine and has highlighted some potentially fruitful avenues for further research.

## Supplementary Information

Below is the link to the electronic supplementary material.

Supplementary Material 1

## Data Availability

The datasets used and/or analysed during the current study is available from the corresponding author on reasonable request.
