# Supplementary material for: Interoception in migraine is characterised by normal accuracy but altered sensibility and behaviour
Source: Sci Rep. 2025 Dec 5;16:1404. doi: 10.1038/s41598-025-31149-0 (PMC12796194; doi:10.1038/s41598-025-31149-0)
Supplement: Supplementary file 1 — Supplementary Material 1 [file 41598_2025_31149_MOESM1_ESM.docx]

Supplementary Information

Principal component analysis

We performed an exploratory principal component analysis (PCA) to understand the factor structure of the questionnaires and their domains. We calculated a correlation matrix and then extracted components with eigenvalue > 1.0. A varimax rotation was conducted on the component loading with Kaiser normalization, and loadings onto domains were considered meaningful if the absolute value was > .40.

**Component structure**

The component analysis revealed 5 components, whose eigenvalues are displayed in supplementary table 1, and matrix of (rotated) loadings in supplementary table 2.

| **Component** | **Eigenvalue** | **% Variance** | **Cumulative % Variance** |
| --- | --- | --- | --- |
| **1** | 4.064 | 27.096 | 27.096 |
| **2** | 2.748 | 18.322 | 45.418 |
| **3** | 1.746 | 11.639 | 57.057 |
| **4** | 1.284 | 8.561 | 65.617 |
| **5** | 1.071 | 7.142 | 72.759 |

**Supplementary table 1: Eigenvalue and % variance explained by the 5 extracted components**

Based on their loadings, the component matrix is as follows and is demonstrated in supplementary Figure 1. Component 1 was a general interoceptive abilities measure, showing no effect of group (F=.268, p=.766). Component 2 related to intolerance of uncertainty, anxiety, worry, and physical sensations associated with these; there was no significant effect of group (F=2.078, p=.135). Component 3 indicated cardiorespiratory sensations and showed no effect of group (F=.113, p=.893). Component 4 indicated depression and showed no effect of group (F=.250, p=.780). Component 5 related to noticing more bodily discomfort, and modifying activities less in response to discomfort, and demonstrated a significant main effect of group (F=4.023, p=.024). Bonferroni corrected post-hoc comparison showed a difference between Ctrl and HF groups (p=.025): that the HF group scored higher than Ctrl group. There were no significant differences between Ctrl and LF groups (p>.99), or between HF and LF groups (p=.130) after correction.

| **Component** | **1** | **2** | **3** | **4** | **5** |
| --- | --- | --- | --- | --- | --- |
| **BPQ: Body Awareness** |  |  | .635 | -.518 |  |
| **BPQ: Supradiaphragmatic Reactivity** |  |  | .810 |  |  |
| **BPQ: Subdiaphragmatic Reactivity** |  |  | .864 |  |  |
| **HADS-A** |  | .789 |  |  |  |
| **HADS-D** |  |  |  | .819 |  |
| **IUS** |  | .626 |  | -.486 |  |
| **MAIA-2: Noticing** | .573 |  |  |  | .587 |
| **MAIA-2: Not distracting** |  |  |  |  | -.837 |
| **MAIA-2: Not worrying** |  | -.711 |  |  |  |
| **MAIA-2: Attention regulation** | .769 |  |  |  |  |
| **MAIA-2: Emotional Awareness** | .818 |  |  |  |  |
| **MAIA-2: Self-regulation** | .844 |  |  |  |  |
| **MAIA-2: Body Listening** | .809 |  |  |  |  |
| **MAIA-2: Trusting** | .788 |  |  |  |  |
| **IDIA** | .408 | .630 |  |  |  |
| **Component interpretation** | **General**  **interoceptive**  **abilities** | **Anxiety** | **Cardiorespiratory / gastrointestinal sensations** | **Depression** | **Noticing and not responding to discomfort** |

**Supplementary table 2: Rotated Component Matrix.** Table of rotated loadings of components onto questionnaire domains. Values exceeding +/- 0.40 are displayed, indicating 'meaningful' associations. Negative values indicate a higher value of that component for an individual indicating a lower score on that questionnaire (domain).


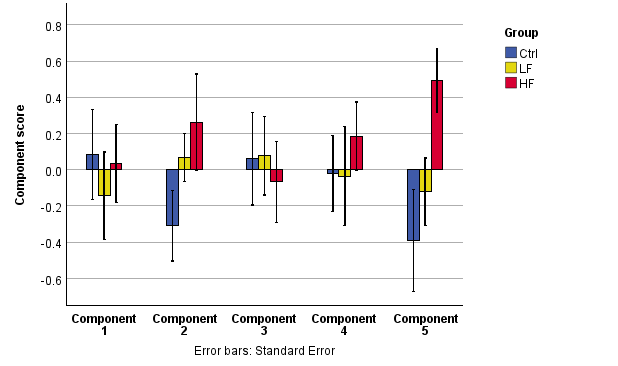


**Supplementary Figure 1: Group comparison of scores on the five PCA components.** Note that higher scores for Component 5 indicate noticing more discomfort and reducing or modifying activity less on account of discomfort.

Correlations

Pearson’s correlation between log-transformed migraine frequency, interoceptive confidence, component 5 and the two MAIA-2 domains loading onto component 5. The correlations were pooled across participants in the LF and HF groups. The frequency data was not normally distributed so the log transform was used to normalise the data.

|  | Log Migraine Frequency | Confidence | Noticing | Not Distracting | Component 5 |
| --- | --- | --- | --- | --- | --- |
| Log Migraine Frequency | -- | -- | -- | -- | -- |
| Confidence | .210 | -- | -- | -- | -- |
| Noticing | .176 | .382* | -- | -- | -- |
| Not Distracting | -.204 | -.042 | -.091 | -- | -- |
| Component 5 | .243 | .137 | .483** | -.851** | -- |

**Supplementary table 3: Correlation between migraine frequency and interoceptive measures and questionnaire data.**

*Note.* For correlations involving questionnaire variables, *N* = 38 (rather than *N* = 39) as one HF participant did not complete all the questionnaires. **p* < .05 (uncorrected). ***p* < .01 (uncorrected).
